# Method for Estimating Amount of Saliva Secreted Using a Throat Microphone

**DOI:** 10.3390/s25123584

**Published:** 2025-06-06

**Authors:** Kai Washino, Ayumi Ohnishi, Tsutomu Terada, Masahiko Tsukamoto

**Affiliations:** Graduate School of Engineering, Kobe University, 1-1 Rokkodaicho, Nada, Kobe 657-8501, Japan; kai-washino@stu.kobe-u.ac.jp (K.W.); ohnishi@eedept.kobe-u.ac.jp (A.O.); tuka@kobe-u.ac.jp (M.T.)

**Keywords:** wearable computing, healthcare, saliva secretion, oral environment

## Abstract

Saliva is an important secretion, and a continued insufficient amount of saliva secreted causes glossitis, stomatitis, and so on. Since the amount of saliva secreted changes daily, adverse effects occur daily. Therefore, it is necessary to constantly measure the amount of saliva secreted and take appropriate measures when it decreases. However, there is no method to constantly measure saliva. We propose a method to estimate the amount of saliva secreted from the sound acquired by a wearable throat microphone. The proposed method uses deep learning to classify whether the sound acquired by the throat microphone is swallowing or not. Based on the swallowing information, the proposed method estimates the amount of saliva secreted. The accuracy of the classification of swallowing was 96.96%. For the estimation of the amount of saliva secreted, the R was 0.600 and MAE was 0.0487.

## 1. Introduction

Saliva is an important secretion, and a continued insufficient amount of saliva secreted is known to cause adverse effects [1,2]. For example, an insufficient amount of saliva secreted causes glossitis, stomatitis, and bad breath [1]. The amount of saliva secreted decreases daily, caused by mental states such as stress [3,4], tension [3,4], and autonomic dysfunction [5].

Therefore, it is necessary to constantly measure the amount of saliva secreted and take appropriate measures when it decreases. In addition, continuous measurement of saliva production is a biomarker for a variety of systemic diseases, since a variety of diseases can also cause decreased saliva production [6]. Furthermore, since the amount of saliva secreted changes depending on mental state [3,4,5], constant measurement of the amount of saliva secreted may enable stress detection and mental care for individuals.

There are several methods to measure the amount of saliva secreted. The methods are summarized in Table 1. Scintigraphy, Magnetic Resonance Imaging (MRI), sonography, and Near-Infrared Spectroscopy (NIRS) are methods to measure salivary gland function and are used during examinations that require specialized knowledge. The cotton method, spitting method, and commercially available methods such as BokaFlo™ [7] and Mucus® [8] can be easily used by anyone. However, existing methods for measuring the amount of saliva secreted cannot be used constantly. The amount of saliva secreted is constantly changing, so it is necessary to measure it continually.

In this paper, we propose a method for estimating the amount of saliva secreted using a wearable throat microphone. To the best of the authors’ knowledge, the proposed method is the first method that can consistently measure the amount of saliva secreted.

The contributions of this study are as follows: We propose a method to continuously measure the amount of saliva secreted using a wearable throat microphone. For the dataset collected in this study, previous methods for classifying swallowing had an accuracy of 89.05%, while we found that methods using deep transfer learning with DenseNet121 achieved an accuracy as high as 96.96%. We proposed a method to estimate the amount of saliva secreted using swallowing frequency, with an R of 0.600 and MAE of 0.0487.

## 2. Related Research

To the best of our knowledge, there is no method to measure the amount of saliva secreted at any time. In this section, we introduce related studies on saliva and swallowing.

### 2.1. Research on Saliva and Swallowing

Saliva is important for the human body, and a decrease in the amount of saliva secreted causes various adverse effects. One of the major functions of saliva is to assist in eating, such as assisting digestion, swallowing, and taste [1,28]. It also has the function of protecting the teeth and oral mucosa, and maintaining oral homeostasis [1,28]. In addition, since the mouth is the starting point of the digestive system, saliva affects the entire digestive system [1]. Thus, saliva is deeply related to the health of the whole body, in addition to the teeth and oral mucosa.

Saliva and swallowing are closely related. Dawes modeled the mechanism of oral clearance [29]. In the model, the amount of saliva secreted in the mouth varies from a minimum of RESID to a maximum of VMAX. Swallowing occurs when the saliva volume in the mouth reaches VMAX. The saliva volume in the mouth after swallowing occurs is RESID. According to this model, the more saliva is secreted, the more swallowing occurs. In fact, Nederkoorn et al. showed that the number of swallows differed depending on the presence or absence of saliva-increasing stimuli [30].

Therefore, it is necessary to detect when the amount of saliva secreted is decreased. Since saliva and swallowing are closely related, the amount of saliva secreted can be estimated using information related to swallowing.

### 2.2. Research on Methods for Measuring the Amount of Saliva Secreted

Several methods have been proposed to measure the amount of saliva secreted. Some methods directly measure the amount of saliva secreted or oral moisture, others measure salivary gland function by imaging salivary rays, and others indirectly estimate the amount of saliva secreted.

Methods that directly measure the amount of saliva secreted include the spitting method, the cotton method, and BokaFlo™. In the spitting method, the subject spits saliva in the mouth into a container. The weight of the saliva spit into the container is used as the amount of saliva secreted. The spitting method is considered to be highly reliable [23], and has been used in various saliva collection studies [24,25]. However, the spitting method requires the subject to spit out saliva. Therefore, the cotton method was proposed to measure the amount of saliva secreted without spitting out saliva [19,20,21]. The cotton method measures the amount of saliva secreted by placing a cotton roll on either the upper or lower part of the tongue and measuring the weight change in the cotton roll. The cotton method is a simple measurement method. BokaFlo™ is another simple method [7]. Saliva can be measured by placing the BokaFlo disposable under the subject’s tongue.

Although we have introduced a method for directly measuring the amount of saliva secreted, methods for measuring oral moisture also exist. Mucus® has been proposed as a method to directly measure oral moisture other than saliva. Mucus® can measure the amount of water in the mucous membranes in 2 s and is a useful instrument, with a small measurement error [8,26,27].

Although direct measurement methods of the amount of saliva secreted and direct oral moisture are reliable, they are difficult to use constantly. The amount of saliva secreted, which changes constantly, needs to be measured constantly, and these methods are not suitable for constant measurement. However, these methods are preferable for judging whether the amount of saliva secreted estimated by the proposed method is correct or not. In this study, we adopted the cotton method as a method to measure the correct amount of saliva secreted.

Medical imaging devices such as scintigraphy, MRI, and sonography have been used to examine salivary gland function [9,10,11,12,14,15,16]. Scintigraphy is an examination method in which a radioactive isotope is injected into the body, and the radiation emitted from the body is measured to produce an image of the inside of the body. Scintigraphy can measure the function of the salivary glands by measuring the radioactivity in the oral cavity [9,10,11]. MRI is an examination in which the body is passed through a tunnel-shaped device that generates a strong magnetic field to produce cross-sectional images of the inside of the body. In previous studies, MRI has been used to evaluate salivary gland function [12,14]. Sonography is an examination method in which high-frequency sound waves are applied to the body, and the bounced sound is used to visualize the inside of the body. Sonography, like MRI and scintigraphy, can evaluate salivary gland function [15]. Gritzmann et al. stated that sonography is the first choice for medical imaging of the salivary glands in terms of detection [16]. These three methods can be used to diagnose salivary gland diseases and salivary secretory capacity. However, scintigraphy requires radioisotopes to be injected into the body, while MRI and sonography require large, specialized machines. Therefore, these methods are not suitable for a device to measure salivary secretion daily, which is the goal of this study.

Several studies have used NIRS to estimate salivary secretion [17,18]. NIRS measures brain function by irradiating the brain with red and near-infrared light, which evaluates oxygenated hemoglobin (HbO_2_) and deoxygenated hemoglobin (HbR). Since HbO_2_ has been shown to correlate with salivary secretion when NIRS is applied to the temple area [17,18], NIRS can be used to estimate salivary secretion. NIRS is one candidate as a method to measure salivary secretion daily. However, brain activity and body movements can significantly affect NIRS measurements, making it difficult to measure salivary secretion in daily life.

Many useful methods have been proposed for measuring the amount of saliva secreted, oral moisture, and for salivary gland evaluation. However, these methods are not suitable for daily measurement. The method proposed in this paper continuously measures the amount of saliva secreted in daily life by detecting swallowing events, which correlate with salivation, and estimating the amount of saliva secreted based on the frequency of swallowing.

### 2.3. Research on a Method for Classifying Swallowing Using a Throat Microphone

A throat microphone can collect throat sounds such as vocalization, coughing, and swallowing. Several methods have been proposed to classify swallowing sounds among the sounds collected by throat microphones.

Several methods for classifying swallowing using a throat microphone have been proposed by Suzuki’s group [31,32,33]. The first method classified swallowing, coughing, and vocalization by counting the number of times the sound exceeded a threshold value [31]. Although their study could classify swallowing sounds, there were many false positives. Jayatilake et al. improved accuracy over a previous study by using Continuous Wavelet Transform (CWT) [32]. Swallowing, coughing, deep breathing, and loud coughing were classified using a scale of 19 amplitudes and a mean zero-crossing number after using CWT for the sounds, with an accuracy of 0.837. Since this estimation accuracy is still insufficient, a method using a deep learning model was proposed to further improve the accuracy [33]. This method used 10.1 ms resolution spectrograms as input and classified whether the sound was swallowing or noise using a Convolutional Neural Network (CNN), achieving an accuracy of 97.3%.

Using the above methods as a reference, this study used a throat microphone to classify swallowing. Although the CNN-based method is the most accurate, a large amount of data are required to create a CNN model with sufficient accuracy. A previous study showed that transfer learning is effective in creating a model with a small amount of data [34]. The main objective of this study was not to classify swallowing but to estimate the amount of saliva secreted using a model that can classify swallowing. To create the most accurate model possible with the least amount of data, a traditional method using a CNN and a method using transfer learning were compared. Based on the comparison results, the model with the highest accuracy was used in the proposed method.

## 3. Method

**Figure 1:**
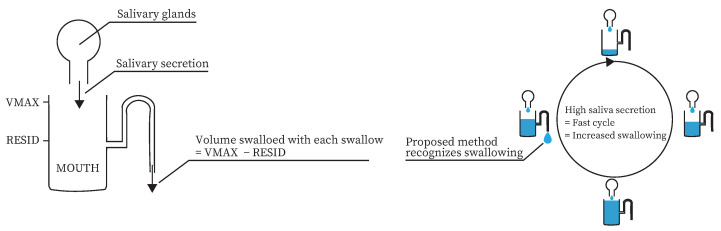
Method for estimating the amount of saliva secreted based on swallowing frequency. Left: similar models of Dawes model; Right: Scheme of the proposed method.

We propose a method for estimating the amount of saliva secreted based on swallowing frequency. Previous studies have shown that the amount of swallowing increases when the amount of saliva secreted varies [30,35]. Dawes modeled the mechanism of oral clearance [29]. A similar model to the Dawes model is shown in Figure 1a. According to this model, the more saliva is secreted, the more swallowing occurs. In addition, several studies have suggested ways to classify swallowing in daily life [31,32,33]. Therefore, swallowing can be used to measure the amount of saliva secreted daily, as shown in Figure 1b.

**Figure 2:**
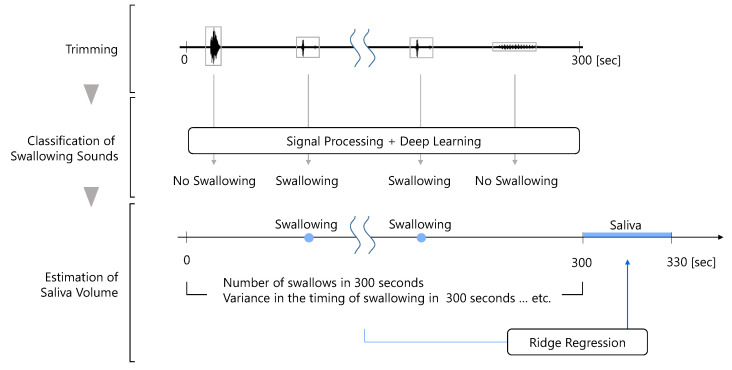
Flow of the proposed method.

The flow of the proposed method for estimating the amount of saliva secreted using a throat microphone is shown in Figure 2. First, the throat microphone acquires throat sounds. Next, whether the throat sound is a swallowing sound or something else is determined. Finally, the amount of saliva secreted is estimated by ridge regression using information such as the number of swallows as feature values.

### 3.1. Acquisition of Throat Sounds

**Figure 3:**
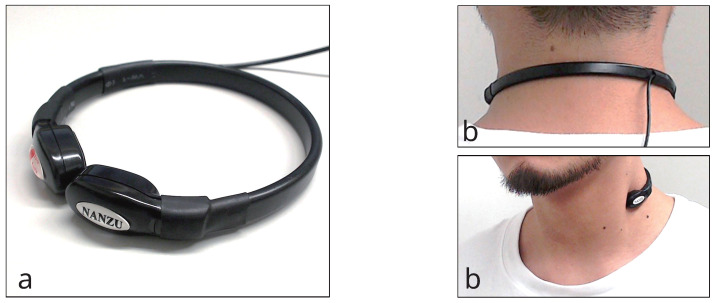
The throat microphone used in the proposed method. (**a**) throat microphone viewed from side; (**b**) Throat microphone worn by the user.

Figure 3 shows the throat microphone (NANZU ELECTRIC Co., Ltd., Shizuoka, Japan, SH-12jK) used in the proposed method. The throat microphone has a sensibility of −40 to 45 dB and a frequency range of 200 Hz to 3 kHz. The sound was acquired at 44.1 kHz.

**Figure 4:**
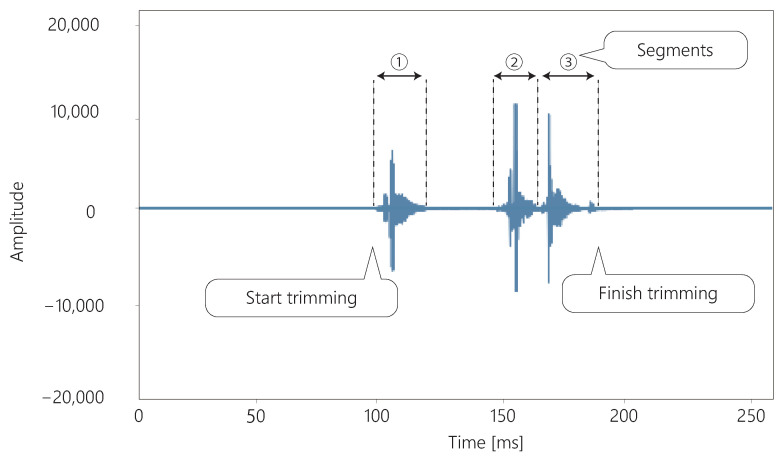
Trimming of the sound acquired by the throat microphone.

The sound acquired by the throat microphone is trimmed, as shown in Figure 4. The sound is trimmed from the moment the sound is produced until the sound is silent for more than 139.5 ms. Swallowing can be divided into one to four segments [33]. Figure 4 shows an example of a three-segment swallow. These segments are never separated by more than 139.5 ms [33]. Therefore, if there is silence for more than 139.5 ms, it is assumed that swallowing is not in progress and the sound is trimmed.

### 3.2. Classification of Swallowing

The proposed method uses deep learning to classify whether the sound acquired by the throat microphone is swallowing or not. The classification of swallowing is based on the method proposed by Kuramoto et al. [33]. First, the sound acquired by the throat microphone is trimmed. The trimmed sound is then converted to a spectrogram through signal processing. Finally, using the spectrogram as data, the deep learning model classifies whether the sound is swallowing or something else.

**Figure 5:**
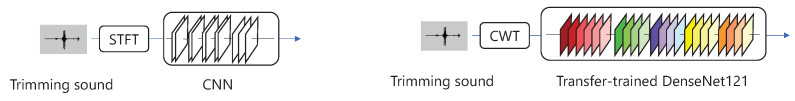
Two methods to classify swallowing. Left: Using STFT and learning with CNN; Right: Using CWT and learning with DenseNet121.

Two methods were candidates for the classification of swallowing, as shown in Figure 5. The first method used Short-Time Fourier Transform (STFT) for the sound in a 10.1 ms window, convolving the data in the time direction using a CNN. The overlap of the STFT was set to 5.05 ms. The CNN consisted of four convolution layers, four pooling layers, and three fully-connected layers. In this method, the activation function was ReLu, the optimizer was Adam, and the loss function was binary cross-entropy. This method is the same as in a previous study [33].

The second method used CWT for the sound, using a complex Gaussian mother wavelet (gaus5), which has been used in a previous study [32]. Next, the acquired spectrogram was converted into 224×224×3 images. The images were used to perform transition learning on DenseNet121 [36] as a spectrogram to classify swallowing. In this method, the optimizer was Adam, and the loss function was binary cross-entropy.

The first method was accurate enough to be used in a previous study [33]. However, it was not clear whether the model would have sufficient accuracy when the amount of data was small. Transfer Learning is effective when the amount of data is small [34]. In addition, CWT has been used to analyze swallowing [32]. Therefore, CWT for sounds and transfer learning for Densely Connected Convolutional Networks (DenseNet) can maintain high accuracy in classifying swallowing, even with a small amount of data.

Both methods are used to reflect the time-directional feature values of sound. Swallowing sounds can be divided into certain segments, and their relationship is a feature of swallowing. [32,37]. Therefore, the classification of swallowing requires the analysis of time series.

### 3.3. Estimation of the Amount of Saliva Secreted from Swallowing

The amount of saliva secreted was estimated by machine learning using feature values calculated from swallowing. The feature values of the proposed method were the number of swallows in 300 s, the number of swallows from 0 to 100 s, from 100 to 200 s, and from 200 to 300 s, and the variance value of the timing of swallowing in 300 s. The learner was ridge regression with α=10.

In this paper, the amount of saliva secreted during the current 30 s was estimated from information about swallowing from 5 min previously up to the current moment. According to previous studies, swallowing occurs an average of one time per minute [38,39]. Therefore, differences in swallowing by the amount of saliva secreted are likely to occur in the range of more than 1 min. However, where this range is too long, there is likely no relationship between swallowing and the amount of saliva secreted. For example, the current amount of saliva secreted may not be high because the amount of swallowing was high 30 min ago. Therefore, the proposed method estimates the amount of saliva secreted based on swallowing over 5 min.

The number of swallows is related to the amount of saliva secreted according to the Dawes model of oral clearance [29]. In the Dawes model, the amount of saliva secreted into the mouth is absorbed into the body through swallowing. In other words, the more saliva is secreted, the greater the number of swallows. In fact, previous studies have shown that there is a relationship between the number of swallows and the amount of saliva secreted [30,35,40]. Therefore, the number of swallows in 5 min is used as a feature.

In addition to the number of swallows in 5 min, the timing of the increase in swallowing frequency is also important. For example, if the number of swallows was high in the first minute of the 5 min, the amount of saliva secreted after 5 min would not be significantly related to the number of swallows. On the other hand, if the swallowing frequency was high in the last minute of the 5 min, the amount of saliva secreted after five minutes would be expected to be high. Therefore, the timing of the increase in the number of swallows is an important indicator.

To obtain the timing of the increase in the number of swallows, the number of swallows from 0 to 100 s, from 100 to 200 s, and from 200 to 300 s were used as feature values. In addition, to checked the variability in the swallowing frequency, and the variance value of the swallowing timing was used as a feature.

**Figure 6:**
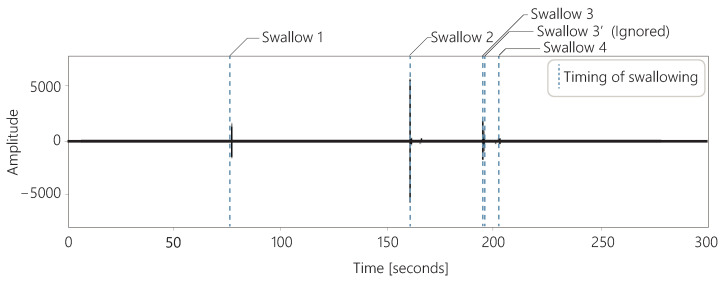
Timing classified as sounds swallowing over 5 min.

Figure 6 shows the sounds acquired with a throat microphone and the timing of swallowing over 5 min for one subject. In these data, the subject swallowed a total of five times. However, the interval between swallow 3 and swallow 3’ shown in the figure was less than 1 s. We observed several cases in which the swallows were perceived as consecutive, as in these data. There are two possible reasons for the occurrence of consecutive swallows: First, the first swallow may have failed, and the user had to swallow again because saliva remained in the mouth. Second, the classification of the swallows using our method may have failed. Whether for first or the second reason, only one of these swallows was considered to be related to the amount of saliva secreted. Therefore, if the swallows were classified as occurring consecutively within 2 s, the second swallow was ignored in our method.

## 4. Experiment 1: Experiment to Confirm the Accuracy of Swallowing Classification

Experiment 1 investigated the accuracy of the proposed method in swallowing classification. The two methods described in Section 3.2 were candidates for the classification of swallowing. The proposed method used the method with the highest accuracy of the two methods. Therefore, the following two methods were evaluated in the first experiment.

Swallowing Classification Method 1: Using STFT and learning with a CNN.
Swallowing Classification Method 2: Using CWT and learning with DenseNet.

### 4.1. Purpose of the Experiment

We experimented to evaluate the accuracy of a method for classifying whether sounds acquired with a throat microphone are swallowing or not. The throat microphone acquired various sounds, such as voice, body movements, and swallowing. The proposed method needs to classify whether the acquired sounds are swallowing or not. Therefore, we prepared swallowing and non-swallowing sounds and evaluated whether they could be classified using the above two methods.

### 4.2. Experiment Design

The accuracy of swallowing classification was evaluated by cross-validating the collected dataset. The dataset consisted of 990 swallowing sounds and 1310 non-swallowing sounds. These datasets were acquired with a throat microphone.

The subjects generated sounds of swallowing and non-swallowing sounds, which were acquired from the throat microphone. The non-swallowing sounds consisted of four types of sounds: vocalization, cough, neck movement, and touching the microphone. The subject wore a throat microphone and generated these sounds within 3 s. We acquired sound data for those 3 s. The sound of swallowing was the sound of the subject swallowing once. The vocalization was a word that the subject generated freely, for example, “a”. Cough was the sound of a single cough by the subject. Neck movement was the sound of the subject moving his neck freely. Touching the microphone was the sound of the subject touching the microphone once.

Cross-validation was performed using a five-fold cross-validation that avoided data bias. This means that the training data consisted of 792 sets of 3 s swallowing sounds and 1048 sets of 3 s non-swallowing sounds, while the test data consisted of 198 sets of swallowing sounds and 262 sets of non-swallowing sounds. The subjects were 18 males in their 20 s.

### 4.3. Results

We compared the accuracy of the STFT and CNN methods and CWT and DenseNet methods on the prepared dataset.

The average accuracy of the five-part cross-validation was 0.89 for Method 1, which performed STFT on the sound and learned with a CNN. The accuracy of each of the folds was 0.92, 0.89, 0.90, 0.86, and 0.88.

The average accuracy of the five-part cross-validation was 0.9696 for Method 2, which used CWT for sound and deep transfer learning with DenseNet. The accuracy of each of the folds was 0.93, 0.93, 0.98, 0.95, and 0.99.

Swallowing Classification Method 2, which performed CWT on the sound and learned with DenseNet, was more accurate than Swallowing Classification Method 1. The proposed method used CWT and DenseNet to classify whether the sound acquired by the throat microphone was swallowing or not.

### 4.4. Discussion

In this study, Swallowing Classification Method 2, which performed CWT on the sound and learned with DenseNet, was more accurate than Swallowing Classification Method 1, an existing method that performed STFT on the sound and learned with a CNN [33]. However, the classification accuracy in the previous study, which collected 3880 data points, was 0.973 [33]. Therefore, Swallowing Classification Method 2 was more accurate in Experiment 1 with a small amount of data; however, it is not yet known which method is more accurate with a larger amount of data.

An additional experiment was also conducted: in Experiment 1, the results included data from the same subject in the training and test data, so the results when the user’s data were not in the training data were not known. Therefore, we recruited three additional male subjects in their 20s who did not participate in the main experiment; the three subjects generated 30 swallowing and 30 non-swallowing sounds to see if we could identify them using the CWT and DenseNet Method. All 2300 datasets used in the experiment were used to train the model. Non-swallowing sounds consisted of four types of sounds: vocalization, cough, neck movement, and touching the microphone.

The result was an accuracy of 0.91. Although it was confirmed that the accuracy dropped a little when the subjects were changed, it can be said that the accuracy was sufficient. Therefore, in the proposed method, the model that was trained on all 2200 datasets was used.

## 5. Experiment 2: Experiment to Confirm the Accuracy of the Estimation of the Amount of Saliva Secreted

In Experiment 2, we evaluated the accuracy of the following method for estimating the amount of saliva secreted.

Saliva Estimation Method: Using ridge regression with swallowing information as feature values.

The proposed method classifies whether the sound acquired by a throat microphone is swallowing or not, and estimates the amount of saliva secreted based on swallowing information for 5 min. Based on the results of Experiment 1, a method using CWT and DenseNet was used to classify swallowing.

The proposed method acquired sounds with a throat microphone for 5 min. As in the first experiment, trimming was automatic in this experiment. When the sound reached more than 10% of the maximum volume, the proposed method automatically started recording. It then finished trimming at the moment that silence began, and when the sound was more than 139.5 ms and less than 10% of the maximum volume. In this way, the 5 min sound was trimmed into several sound segments. The trimmed sounds were classified as swallowing or not swallowing. The proposed method estimated the amount of saliva secreted based on swallowing information, such as the number of swallows in 5 min.

**Figure 7:**
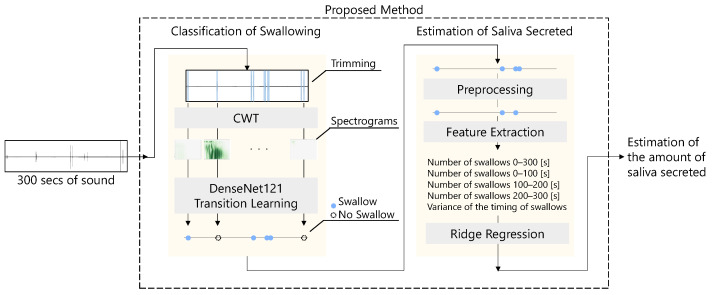
The input and output data for each flow.

The input and output data for each flow are shown in Figure 7. First, 5 min of sound acquired with a throat microphone was trimmed and divided into multiple sound data. The divided sound data were converted into spectrograms using CWT. As described in Section 3.2, a complex Gaussian mother wavelet (gaus5) was used in the CWT. From the converted images, DenseNet121 transfer learning was used to identify whether the sound was swallowing or not. From the identification results, five features were obtained, including the number of swallows and the timing of the swallowing. Based on these features, the amount of saliva secreted was estimated using ridge regression.

We experimented to evaluate the accuracy of the method for estimating the amount of saliva secreted using swallowing information as feature values. The subjects were 17 males in their 20s, the same individuals as in Experiment 1.

### 5.1. Experiment Design

One trial of Experiment 2 consisted of 5 min of swallowing acquisition time and 30 s measurement for the amount of saliva secreted. Since each subject performed 5 trials, 85 data points (5×17) were obtained. We evaluated the 85 data points with leave-one-out cross-validation. In this evaluation, 84 out of 85 datasets were used for learning. The resulting or learning model was used to estimate saliva secretion for one dataset. This was repeated 85 times to calculate the measured values for all the data.

**Figure 8:**
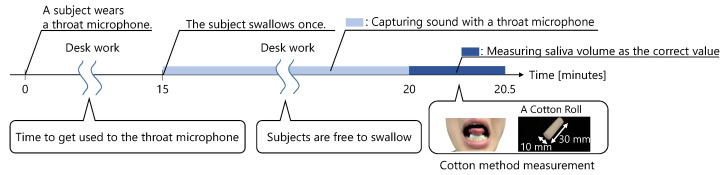
A trial of Experiment 2.

A trial is shown in Figure 8. First, the subject wore a throat microphone and started working at a desk. At least 15 min after wearing the microphone, the subject swallowed. After the subject swallowed, the first trial started. The throat sounds were captured by the throat microphone for 5 min immediately after swallowing. Then, 5 min later, the amount of saliva secreted was measured. While wearing the throat microphone, the subjects were not allowed to speak, eat, drink, or leave their seat until the amount of saliva secreted was measured.

We used a cotton method to measure the amount of saliva secreted [19,20]. Figure 8 shows how subjects measured the amount of saliva secreted using the cotton method. In this method, a cotton roll, approximately 10 mm wide and 30 mm long, was placed under the subject’s tongue, with the mouth lightly closed. After 30 s, the cotton roll was removed. Then, the amount of saliva secreted was measured using the change in the weight of the cotton roll. Before the 30 s measurement, the subject swallowed any saliva remaining in the mouth. During the 30 s measurement, the subject was told to not move his face nor swallow.

### 5.2. Result

The proposed method estimated the amount of saliva secreted based on 5 min swallowing information. The correct amount of saliva secreted was measured by the cotton method. From the experiment, the Correlation Coefficient (R) of the proposed method was 0.600, and the Mean Absolute Error (MAE) was 0.0487. MAE represents the mean of the error between the measured and estimated values of saliva secretion, and is obtained by the following equation, where $x_{i}$ is the measured value, $y_{i}$ is the predicted value, and *n* is the total number of data points.

$$MAE=\frac{1}{n}\sum_{i=1}^{n}\left|y_{i}-x_{i}\right|$$

**Figure 9:**
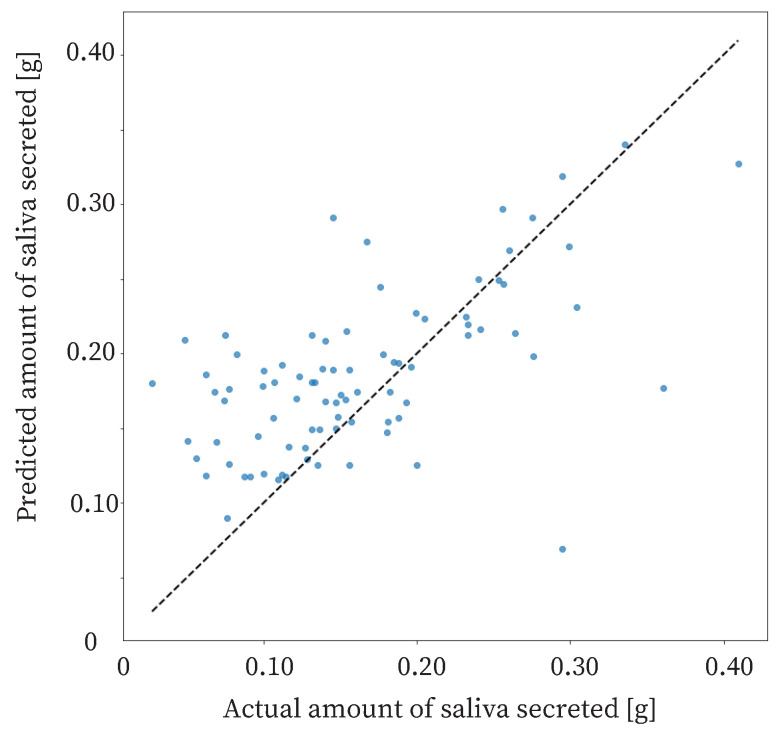
Estimated results of the amount of saliva secreted.

The results are shown in Figure 9.

### 5.3. Discussion

The R of the proposed method was 0.600, and the MAE was 0.0487. A reasonable accuracy for the proposed method is an MAE of 0.07 g or less. Therefore, the proposed method was sufficiently accurate. The proposed method should detect large changes in salivary secretion. In other words, the accuracy of the method should be able to detect three types of changes in saliva production: an extreme decrease in saliva production due to stress, a normal state, and an extreme increase in saliva production due to stimulation. Therefore, the proposed method distinguishes between an extreme decrease in saliva production, the normal state, and an extreme increase in saliva production. A previous study showed that the amount of saliva secreted is approximately 0.24 g in the normal, non-stimulated state, and approximately 0.44 g when saliva secretion is increased by odor stimulation [41]. In addition, another previous study showed that the threshold for insufficient salivary secretion is 0.10 g [27]. The difference between insufficient and normal saliva production is approximately 0.14 g. The difference between normal and increased saliva production is approximately 0.20 g. Therefore, the estimation accuracy required for the method is 0.07 g, which can be divided into two parts for a difference of approximately 0.14 g.

There is a possibility that a saliva volume estimation method with sufficient accuracy could be realized by improving the proposed method. We consider that there are four major ways to improve the proposed method. The first is to increase the amount of data. We believe that increasing the amount of data would increase the accuracy of machine learning and improve the accuracy of salivary volume estimation. The second is to increase the swallowing sound acquisition time from 5 to 10 min, for example. Data with low salivary secretion tended to have low swallowing frequency. If saliva production was very low, about 0.05 g, the number of swallows in 5 min was one or two, and if saliva production was a little low, about 0.1 g, the number of swallows was also one or two. Thus, it is possible that the short 5 min time period did not measure the fine differences in the data between very low and slightly low salivary production. Therefore, a method that acquires data for 10 to 15 min and estimates salivary production would be a good candidate. The third method is to perform calibration for each user. First, as a calibration, the user could measure the number of swallows and the correct salivary volume for 5 min. This result would be compared with the number of swallows in daily life to estimate the amount of saliva secreted. For estimation, the method would be to estimate the amount of saliva secreted based on whether the number of swallows has increased or decreased, based on this calibrated value. The fourth method is to use an additional sensor. Salivary secretion is affected by mental changes. Therefore, it may be possible to measure salivary secretion by using a heart rate sensor to measure mental changes, and then using the measurement results and swallowing frequency to measure the amount of saliva secreted.

**Figure 10:**
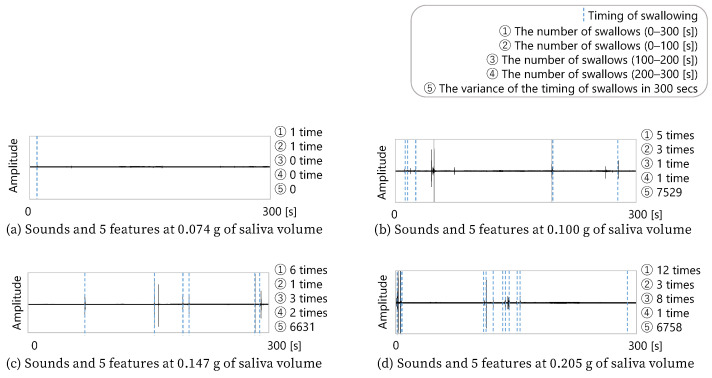
Examples of acquired sounds and saliva volume for four subjects.

Figure 10 shows four combinations of five minutes of sound data, the features obtained from the sound data, and the amount of saliva secreted by the subject after the five min of sound acquisition. Since these four data examples were extracted from the total data, this cannot be said for all the data, but it can be seen that the more frequently the subject swallowed, the more saliva was secreted. In addition, b and c in the figure show that the difference in the number of swallows between b and c was only one. However, c had more swallows in the second half, while b had more swallows in the first half. c, who swallowed more often in the second half, secreted more saliva. Therefore, these four data examples show that the number of swallows and the timing of swallowing were related to the amount of saliva secreted.

**Figure 11:**
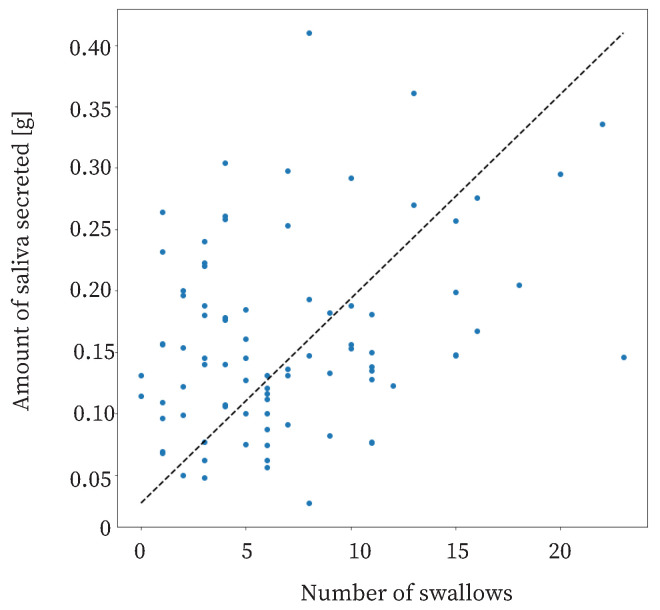
Relationship between the number of swallows and the amount of saliva secreted.

The proposed method is based on previous studies showing that swallowing increases as the amount of saliva secreted increases [30,35,40]. The relationship between the amount of saliva secreted and swallowing in this study is shown in Figure 11. The coefficient of correlation was approximately 0.31. This means that there was a weak correlation between the amount of saliva secreted and the number of swallows. Therefore, it was useful to use the number of swallows as a feature.

**Figure 12:**
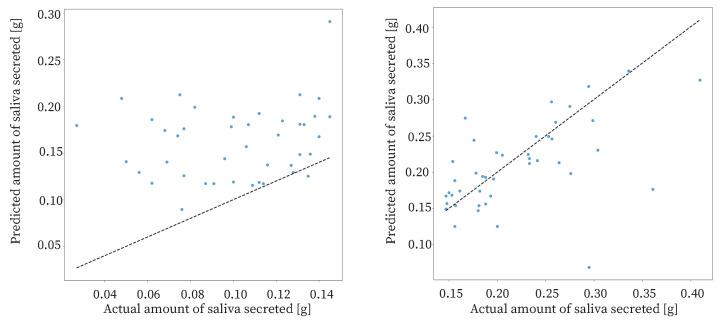
Estimation results for high and low amounts of saliva secreted data. Left: Half with a low amount of saliva secreted; Right: Half with a high amount of saliva secreted.

The study had 85 datasets. The data were sorted based on the amount of saliva secreted and divided into two halves: one half with a high amount of saliva secreted and the other half with a low amount of saliva secreted. The results of the proposed method for each dataset are shown in Figure 12. For the data with a low amount of saliva secreted, the MAE was approximately 0.061. For the data with a high amount of saliva secreted, the MAE was approximately 0.037. The estimation accuracy for data with a high amount of saliva secreted was higher than that for the data with a low amount of saliva secreted. Therefore, the proposed method was effective when the amount of saliva secreted was high. The reason for the low accuracy of estimation in the case of a low amount of saliva secreted may be that swallowing is less frequent when the amount of saliva secreted is low.

**Figure 13:**
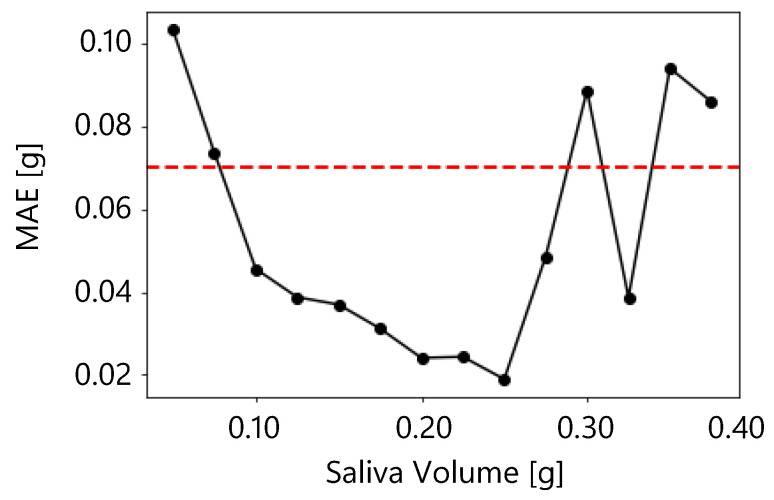
Moving average of MAE for salivary secretion.

A reasonable accuracy for the proposed method is an MAE of 0.07 g or less, and an accuracy of 0.07 g or less was obtained in the present results when the salivary secretion was between 0.075 g and 0.275 g. Figure 13 shows the moving average of MAE for salivary secretion. In the moving average, the window size was 0.05 g and the overlap was 0.025 g. The figure shows that the MAE was less than 0.07 g when the salivary volume was between 0.075 g and 0.275 g. Therefore, the proposed method was sufficiently accurate between 0.075 g and 0.275 g.

**Figure 14:**
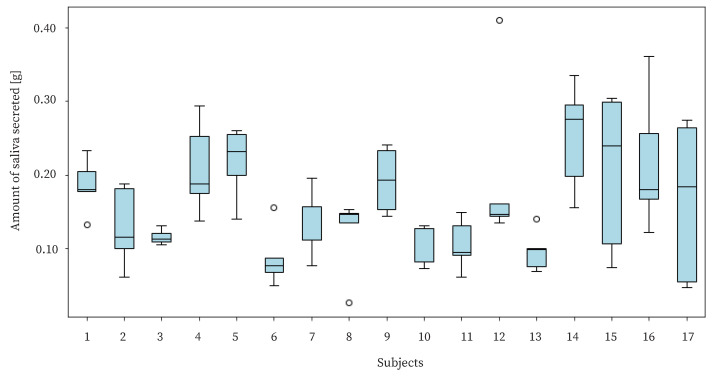
The amount of saliva secreted for each subject.

The purpose of this study is to benefit users by live-logging the amount of saliva secreted. Therefore, it is necessary to detect changes in the amount of saliva secreted by individual users. In the experiment, we needed to check whether there was a difference in the amount of saliva secreted by each user and whether the proposed device could detect such a difference. The amount of saliva secreted by each user is shown in Figure 14. The figure shows a large variation in the amount of saliva secreted for each subject. The standard deviation of the amount of saliva secreted for each individual was approximately 0.0583, and the standard deviation of the amount of saliva secreted by the whole group was approximately 0.0779. Since there was not a large difference between the variance value of the amount of saliva secreted within each individual and that of the total amount of saliva secreted, it can be said that the proposed method estimated the difference using the amount of saliva secreted within an individual.

## 6. Limitations

In this study, we proposed a method for estimating the amount of saliva secreted by using swallowing information for 5 min as feature values. The accuracy of the proposed method was evaluated on male subjects their 20s. However, it is necessary to investigate other feature values, other learners, other genders and age groups, and various situations in daily life.

The feature values used in the proposed method were the number of swallows in 5 min, the variance value of the timing of swallowing in 5 min, the number of swallows from 0 to 100 s, the number of swallows from 100 to 200 s, and the number of swallows from 200 to 300 s. In addition, there are several other possible feature values. There are also other candidates for the feature values used in the proposed method.

For example, there could be the number of swallows per 10 min. The results in this paper were obtained when the frequency of swallowing was measured for 5 min. However, the accuracy of salivary volume estimation at longer measurement times may be higher, as mentioned in Section 5.3. Therefore, the results in this paper have the limitation that the results were for a measurement time of 5 min.

In Experiment 1, a 3 s sound was classified as swallowing or not swallowing with approximately 0.9696 accuracy, but it is not known whether the five minutes of data could be successfully classified as swallowing. The 5 min of sound data were automatically trimmed, and the frequency of swallowing was calculated; identification of swallows for the 5 min sound data may not be successful in two respects: first, whether the trimming is carried out well, and second, whether the swallows themselves are identified well. The trimming of the swallows was based directly on the method used in the previous study [32]. We confirmed that the trimming for the 3 s data worked well, but since the 5 min data were natural-life data, we cannot confirm whether the trimming was successful. In addition, we do not know whether the swallowing discrimination for the experimental data was reliable, since there are no correct swallowing timing data.

In addition, although the subjects were not allowed to speak while working at the desk, they were able to move freely to some extent, which may have reduced the accuracy of the classification. Therefore, it is necessary to investigate whether swallowing can be classified based on 5 min of data.

We could also consider using the amount of saliva secreted swallowed as a feature value. The amount of saliva secreted in one swallow was approximately 0.30 mL, and the SD was approximately 0.16 mL [42]. In other words, the amount of saliva secreted swallowed was variable. If the amount of saliva secreted swallowed was known, the number of swallows and the amount of saliva swallowed could be used to estimate the amount of saliva secreted more precisely. Several methods have been proposed to estimate swallowed items and swallowing volume from swallowing information [43,44]. In addition, since it is known that swallowing volume is related to the length of swallowing time [45], the length of swallowing time was assumed to reflect the swallowing volume and was used as a candidate feature.

Although ridge regression was used as the learner in the proposed method, other learners need to be considered. Since only 85 datasets were obtained in this study, it was not possible to create validation data and adjust the hyperparameters and the learner. A Random Forest Regressor, gradient boosting regression, and SVR should be considered.

We recruited men for the experiment. However, Percival et al. showed that the amount of saliva secreted differs between males and females [46]. Therefore, it is necessary to conduct the same experiment not only on males, but also on females.

All subjects were in their 20s. However, Fenoli et al. showed that the amount of saliva secreted decreases with age [47]. Therefore, it is necessary to confirm whether the proposed method can estimate the amount of saliva secreted by the elderly as well.

## 7. Conclusions

In this study, we proposed a method for constantly estimating the amount of saliva secreted from swallowing. The proposed method classifies swallowing using continuous wavelet transform and DenseNet transfer learning on the sound acquired by a throat microphone. In addition, the proposed method used ridge regression to estimate the amount of saliva secreted using swallowing frequency for 5 min as a feature value. Swallowing was classified as approximately 96.96%. The estimated amount of saliva secreted was approximately 0.600 for R, and approximately 0.0487 for MAE.

## Figures and Tables

**Table 1:** Existing methods to measure the amount of saliva secreted.

Method Name	Can Be Used Without Expertise	Can Be Measured at All Times	References
Scintigraphy	No	No	[9,10,11]
MRI	No	No	[12,13,14]
Sonography	No	No	[15,16]
NIRS	No	No	[17,18]
Cotton Method	Yes	No	[19,20,21,22]
Spitting Method	Yes	No	[23,24,25]
BokaFlo™	Yes	No	[7]
Mucus®	Yes	No	[8,26,27]

## Data Availability

The research data are not publicly available due to the restrictions from the Ethics Committee of the Graduate School of Engineering, Kobe University.

## References

[1] Edgar W.M., O’Mullane D., Dawes C. (2004). Saliva and Oral Health.

[2] Robati B.F., Mirhedayati S.E., Mirhedayati S.A. (2024). Exploring Connections Between Diet, Saliva Production, and Oral Health. Int. J. New Find. Health Educ. Sci..

[3] Thie N.M., Kato T., Bader G., Montplaisir J.Y., Lavigne G.J. (2002). The Significance of Saliva during Sleep and the Relevance of Oromotor Movements. Sleep Med. Rev..

[4] Imamura T.K., Yoshino Y., Yamachika S., Ishii H., Watanabe N.Y., Inoue H., Nakagawa Y. (2012). Inhibition of Pilocarpine-induced Saliva Secretion by Adrenergic Agonists in ICR Mice. Clin. Exp. Pharmacol. Physiol..

[5] Bagheri H., Damase-Michel C., Lapeyre-Mestre M., Cismondo S., O’Connell D., Senard J.M., Rascol O., Montastruc J.L. (1999). A Study of Salivary Secretion in Parkinson’s Disease. Clin. Neuropharmacol..

[6] Proctor G., Shaalan A. (2021). Disease-Induced Changes in Salivary Gland Function and the Composition of Saliva. J. Dent. Res..

[7] Fallon B.S., Chase T.J., Cooke E.M., Ghazitabatabaei A., Naylor N.O., Cutshall J.J., Trump B.G., Weller M.L. (2021). The Use of BokaFlo™ Instrument to Measure Salivary Flow. BMC Oral Health.

[8] Fukushima Y., Yoda T., Araki R., Sakai T., Toya S., Ito K., Funayama S., Enoki Y., Sato T. (2017). Evaluation of Oral Wetness Using an Improved Moisture-checking Device for the Diagnosis of Dry Mouth. Oral Sci. Int..

[9] Aung W., Murata Y., Ishida R., Takahashi Y., Okada N., Shibuya H. (2001). Study of Quantitative Oral Radioactivity in Salivary Gland Scintigraphy and Determination of the Clinical Stage of Sjögren’s Syndrome. J. Nucl. Med..

[10] Van den Akker H., Sokole E.B., Van der Schoot J. (1976). Origin and Location on the Oral Activity in Sequential Salivary Gland Scintigraphy with 99mTc-Pertechnetate. J. Nucl. Med. Off. Publ. Soc. Nucl. Med..

[11] Faria R.A., Jaguar G.C., Lima E.N.P. (2024). Parametric Imaging in Salivary Gland Scintigraphy. Nucl. Med. Commun..

[12] Simon-Zoula S.C., Boesch C., De Keyzer F., Thoeny H.C. (2008). Functional Imaging of the Parotid Glands Using Blood Oxygenation Level Dependent (BOLD)-MRI at 1.5 T and 3 T. J. Magn. Reson. Imaging Off. J. Int. Soc. Magn. Reson. Med..

[13] Guévelou J.L., Palard-Novello X., Kammerer E., Baty M., Perazzi M., Larnaudie A., de Crevoisier R., Castelli J. (2024). Assessment and Prediction of Salivary Gland Function after Head and Neck Radiotherapy: A Systematic Review. Cancer Med..

[14] Patel R.R., Carlos R.C., Midia M., Mukherji S.K. (2004). Apparent Diffusion Coefficient Mapping of the Normal Parotid Gland and Parotid Involvement in Patients with Systemic Connective Tissue Disorders. Am. J. Neuroradiol..

[15] Martinoli C., Derchi L., Solbiati L., Rizzatto G., Silvestri E., Giannoni M. (1994). Color Doppler Sonography of Salivary Glands. AJR Am. J. Roentgenol..

[16] Gritzmann N., Rettenbacher T., Hollerweger A., Macheiner P., Hübner E. (2003). Sonography of the Salivary Glands. Eur. Radiol..

[17] Sato H., Obata A.N., Moda I., Ozaki K., Yasuhara T., Yamamoto Y., Kiguchi M., Maki A., Kubota K., Koizumi H. (2011). Application of Near-infrared Spectroscopy to Measurement of Hemodynamic Signals Accompanying Stimulated Saliva Secretion. J. Biomed. Opt..

[18] Matsumoto T., Saito K., Nakamura A., Saito T., Nammoku T., Ishikawa M., Mori K. (2012). Dried-Bonito Aroma Components Enhance Salivary Hemodynamic Responses to Broth Tastes Detected by Near-Infrared Spectroscopy. J. Agric. Food Chem..

[19] Takahashi F., Koji T., Morita O. (2006). Oral Dryness Examinations: Use of an Oral Moisture Checking Device and a Modified Cotton Method. Prosthodont. Res. Pract..

[20] Ichikawa K., Sakuma S., Yoshihara A., Miyazaki H., Funayama S., Ito K., Igarashi A. (2011). Relationships between the Amount of Saliva and Medications in Elderly Individuals. Gerodontology.

[21] Kakinoki Y. (2015). Disease Condition and Treatment of Dry Mouth. Jpn. Prosthodont. Soc..

[22] Strazdins L., Meyerkort S., Brent V., D’Souza R.M., Broom D.H., Kyd J.M. (2005). Impact of Saliva Collection Methods on sIgA and Cortisol Assays and Acceptability to Participants. J. Immunol. Methods.

[23] Navazesh M., Christensen C. (1982). A Comparison of Whole Mouth Resting and Stimulated Salivary Measurement Procedures. J. Dent. Res..

[24] Justino A.B., Teixeira R.R., Peixoto L.G., Jaramillo O.L.B., Espindola F.S. (2017). Effect of Saliva Collection Methods and Oral Hygiene on Salivary Biomarkers. Scand. J. Clin. Lab. Investig..

[25] Dawes C. (1987). Physiological Factors Affecting Salivary Flow Rate, Oral Sugar Clearance, and the Sensation of Dry Mouth in Man. J. Dent. Res..

[26] Takahashi F., Koji T., Morita O. (2005). The Usefulness of an Oral Moisture Checking Device (Moisture Checker for Mucus). Nihon Hotetsu Shika Gakkai Zasshi.

[27] Takahashi F., Takahashi M., Toya S., Koji T., Morita O. (2006). Clinical Usefulness of an Oral Moisture Checking Device (Mucus®). Prosthodont. Res. Pract..

[28] Mese H., Matsuo R. (2007). Salivary Secretion, Taste and Hyposalivation. J. Oral Rehabil..

[29] Dawes C. (1983). A Mathematical Model of Salivary Clearance of Sugar from the Oral Cavity. Caries Res..

[30] Nederkoorn C., Smulders F.T., Jansen A. (1999). Recording of Swallowing Events using Electromyography as a Non-Invasive Measurement of Salivation. Appetite.

[31] Nagae M., Suzuki K. (2011). A Neck Mounted Interface for Sensing the Swallowing Activity Based on Swallowing Sound. Proceedings of the 2011 Annual International Conference of the IEEE Engineering in Medicine and Biology Society.

[32] Jayatilake D., Ueno T., Teramoto Y., Nakai K., Hidaka K., Ayuzawa S., Eguchi K., Matsumura A., Suzuki K. (2015). Smartphone-Based Real-Time Assessment of Swallowing Ability from the Swallowing Sound. IEEE J. Transl. Eng. Health Med..

[33] Kuramoto N., Ichimura K., Jayatilake D., Shimokakimoto T., Hidaka K., Suzuki K. (2020). Deep Learning-Based Swallowing Monitor for Realtime Detection of Swallow Duration. Proceedings of the 2020 42nd Annual International Conference of the IEEE Engineering in Medicine & Biology Society (EMBC).

[34] Tan C., Sun F., Kong T., Zhang W., Yang C., Liu C. (2018). A Survey on Deep Transfer Learning. Proceedings of the Artificial Neural Networks and Machine Learning–ICANN 2018: 27th International Conference on Artificial Neural Networks.

[35] Kapila Y.V., Dodds W.J., Helm J.F., Hogan W.J. (1984). Relationship Between Swallow Rate and Salivary Flow. Dig. Dis. Sci..

[36] Huang G., Liu Z., Van Der Maaten L., Weinberger K.Q. Densely Connected Convolutional Networks. Proceedings of the IEEE Conference on Computer Vision and Pattern Recognition.

[37] Hong-Seop K. (2014). Understanding of Xerostomia and Strategies for the Development of Artificial Saliva. Chine J. Dent. Res..

[38] Lear C.S., Flanagan J., Moorrees C. (1965). The Frequency of Deglutition in Man. Arch. Oral Biol..

[39] Dent J., Dodds W.J., Friedman R.H., Sekiguchi T., Hogan W.J., Arndorfer R.C., Petrie D.J. (1980). Mechanism of Gastroesophageal Reflux in Recumbent Asymptomatic Human Subjects. J. Clin. Investig..

[40] Rudney J., Ji Z., Larson C. (1995). The Prediction of Saliva Swallowing Frequency in Humans from Estimates of Salivary Flow Rate and the Volume of Saliva Swallowed. Arch. Oral Biol..

[41] Washino K., Ohnishi A., Terada T., Tsukamoto M. (2025). Evaluation of Salivation Promotion by Odor, Pressure, and Thermal Stimulus for Designing Wearable Device to Increase Salivation. Sci.

[42] Lagerlof F., Dawes C. (1984). The Volume of Saliva in the Mouth before and after Swallowing. J. Dent. Res..

[43] Yamada Y., Saito T., Kawasaki S., Ikeda D., Katagiri M., Nishimura M., Mineno H. (2017). A Deep-Learning-Based Method of Estimating Water Intake. Proceedings of the 2017 IEEE 41st Annual Computer Software and Applications Conference (COMPSAC).

[44] Sazonov E.S., Makeyev O., Schuckers S., Lopez-Meyer P., Melanson E.L., Neuman M.R. (2009). Automatic Detection of Swallowing Events by Acoustical Means for Applications of Monitoring of Ingestive Behavior. IEEE Trans. Biomed. Eng..

[45] Molfenter S.M., Steele C.M. (2013). Variation in Temporal Measures of Swallowing: Sex and Volume Effects. Dysphagia.

[46] Percival R., Challacombe S., Marsh P. (1994). Flow Rates of Resting Whole and Stimulated Parotid Saliva in Relation to Age and Gender. J. Dent. Res..

[47] FenolI-Palomares C., Muñoz-Montagud J., Sanchiz V., Herreros B., Hernández V., Mínguez M., Benages A. (2004). Unstimulated Salivary Flow Rate, PH and Buffer Capacity of Saliva in Healthy Volunteers. Rev. Esp. Enfermedades Dig..

